# Proteome-Wide Profiling Using Sample Multiplexing of a Human Cell Line Treated with Cannabidiol (CBD) and Tetrahydrocannabinol (THC)

**DOI:** 10.3390/proteomes11040036

**Published:** 2023-11-02

**Authors:** Morteza Abyadeh, Vivek Gupta, Xinyue Liu, Valentina Rossio, Mehdi Mirzaei, Jennifer Cornish, Joao A. Paulo, Paul A. Haynes

**Affiliations:** 1 ProGene Technologies Pty Ltd., Macquarie Park, NSW 2113, Australia; abyadehm@vcu.edu; 2 Macquarie Medical School, Faculty of Medicine, Health and Human Sciences, Macquarie University, Sydney, NSW 2109, Australia; vivek.gupta@mq.edu.au (V.G.); mehdi.mirzaei@mq.edu.au (M.M.); 3 Department of Cell Biology, Harvard Medical School, Boston, MA 02115, USA; xinyue_liu@hms.harvard.edu (X.L.); valentina_rossio@hms.harvard.edu (V.R.); joao_paulo@hms.harvard.edu (J.A.P.); 4 School of Psychological Sciences, Faculty of Medicine, Health and Human Sciences, Macquarie University, Sydney, NSW 2109, Australia; jennifer.cornish@mq.edu.au; 5 School of Natural Sciences, Macquarie University, North Ryde, NSW 2109, Australia

**Keywords:** tandem mass tags, TMTpro, real-time database searching, unfolded protein response, high-field asymmetric ion mobility spectrometry, quantitative proteomics, cannabidiol, Delta-9-tetrahydrocannabinol, mitochondrial dysfunction, endoplasmic reticulum stress

## Abstract

Cannabis has been used historically for both medicinal and recreational purposes, with the most notable cannabinoids being cannabidiol (CBD) and tetrahydrocannabinol (THC). Although their therapeutic effects have been well studied and their recreational use is highly debated, the underlying mechanisms of their biological effects remain poorly defined. In this study, we use isobaric tag-based sample multiplexed proteome profiling to investigate protein abundance differences in the human neuroblastoma SH-SY5Y cell line treated with CBD and THC. We identified significantly regulated proteins by each treatment and performed a pathway classification and associated protein–protein interaction analysis. Our findings suggest that these treatments may lead to mitochondrial dysfunction and induce endoplasmic reticulum stress. These data can potentially be interrogated further to investigate the potential role of CBD and THC in various biological and disease contexts, providing a foundation for future studies.

## 1. Introduction

Cannabis, commonly known as marijuana, is a generic term for *Cannabis sativa* or *Cannabis indica* plants that contain multiple compounds that are collectively known as cannabinoids, and has been used since ancient times for medicinal and recreational purposes [1]. Recent decades have seen a growing interest in studying the therapeutic properties of cannabis, driven by their observed beneficial effects against several diseases, increasing social acceptance, and modifications to legislation around cannabis use policies [2]. Much of the interest in, and published studies on, the therapeutic and toxic effects of cannabis revolve around two main types of cannabinoids: cannabidiol (CBD) and delta-9-tetrahydrocannabinol (THC) [3]. However, the mechanisms underlying their therapeutic effects are poorly defined.

Both THC and CBD interact with G protein-coupled receptors (GPCRs), including cannabinoid receptor 1 (CBR1), cannabinoid receptor 2 (CBR2), ion channels, and various nuclear receptors; their effects are believed to be mainly due to their binding with CBR1 and CBR2. CBD and THC are structurally very similar but have different binding affinities for cannabinoid receptors, which result in different biological and pharmacological properties [4]. THC is the primary psychoactive compound in cannabis and is considered to be toxic, while CBD is a non-psychoactive cannabinoid with multiple therapeutic properties [5,6]. A wealth of evidence is available that supports the beneficial effects of THC and CBD in different diseases including multiple neurodegenerative disorders, infectious diseases, cardiovascular diseases (CVDs), autoimmune diseases, fatty liver disease, and cancer [7,8]. Conversely, toxic and adverse effects of these compounds have also been reported in different cell and animal model studies [9,10,11].

Despite considerable studies on the therapeutic effects of cannabis derivatives and mechanisms underlying their effects, the compounds have found limited translational applications. There are currently four prescription drugs derived from or related to cannabis which have been approved by the US Food and Drug Administration. Epidiolex, which contains CBD, is approved for the treatment of seizures associated with tuberous sclerosis complex. Cesamet (nabilone), Marinol (dronabinol), and Syndros (dronabinol), are all THC-containing and are used in treatments to prevent weight loss and increase appetite in AIDS patients, as well as treatment of persistent nausea and vomiting associated with chemotherapy regimens [12,13].

The use of THC and CBD has been restricted by two key challenges: the lack of information about the mechanisms of action underlying their therapeutic effects, and the limited research regarding their safety. Therefore, there is an urgent need to carry out further studies to comprehensively investigate the potential mechanisms underlying the therapeutic or toxic effects of these molecules.

High throughput technologies such as genomics, transcriptomics, proteomics, lipidomics, and metabolomics have opened new windows for drug development [14]. Quantitative proteomics has benefited greatly from the development of isotopic labeling techniques including isotope tagging for relative and absolute quantitation (iTRAQ) [15,16] and tandem mass tags (TMT) [2]. A growing number of molecular studies investigating the molecular targets of CBD and THC have been presented, although these include only a modest number of proteome-wide abundance profiling datasets from limited biological samples, such as primary human keratinocytes, astrocytes, and animal brain samples [17].

Many of the previous proteomics studies on the cellular effects of cannabis products are focused on the brain, since the central nervous system is the primary target of action of cannabis [18]. Associations of cannabis with numerous mental illnesses, including schizophrenia, have also been reported [19]. A study focused on olfactory neuroepithelium cells from the nasal cavity of cannabis users identified differentially expressed proteins related to apoptosis, cell proliferation, and cytoskeletal remodeling [20]. In an interesting study focused on olfactory neuroepithelium cells from schizophrenia patients who were also cannabis users, distinct impacts of cannabis were identified on proteins related to immune system functions and protein metabolism [21]. There are also previous studies on investigating changes in the urinary proteome of cannabis users, with the urothelium known to be a major site of action of cannabis and similar related compounds [22]. Changes have been observed in proteins related to immune response, lipid metabolism, and inflammatory activity in the urinary proteome of cannabis users when compared to healthy controls [23].

Here, we have used sample multiplexing (TMTpro16), high-field asymmetric ion mobility spectrometry (FAIMS), and real-time database searching (RTS) to explore proteome alteration in the SH-SY5Y cell line following treatments with CBD and THC. The results of these experiments shed light on the mechanisms corresponding to the therapeutic and toxic cellular effects of these cannabinoids.

## 2. Materials and Methods

### 2.1. Materials

Mass spectrometry grade organic solvents and HPLC grade water were from J.T. Baker (Center Valley, PA, USA), mass spectrometry grade trypsin was from Pierce Biotechnology (Rockford, IL, USA), and LysC was from Wako Chemicals (Richmond, VA, USA). Antibiotics including streptomycin and penicillin were from Gibco (Waltham, MA, USA), and cultures of SH-SY5Y cells were purchased from ATCC (Manassas, VA, USA). Dulbecco’s modified Eagle’s medium (DMEM), which was supplemented with 10% fetal bovine serum (FBS) was from Life Technologies (Waltham, MA, USA). Tandem mass tag (TMTpro) isobaric labeling reagents for quantitative proteomics were purchased from Thermo Fisher Scientific (Waltham, MA, USA).

### 2.2. Treatment of Cells with Compounds

Cellular stocks with low passages were used to initiate cultures that were grown in a 5% carbon dioxide incubator at 37 °C. SH-SY5Y cell cultures were grown in DMEM supplemented with 10% FBS, along with 50 µL/mL streptomycin (1000 µg/mL), 50 µL/mL penicillin (1000 units/mL), and additional glucose (4500 mg/L)/pyruvate (1 mM). The growth media was removed, and the cells were thoroughly washed with phosphate-buffered saline, once the initial plates had grown to approximately 50% confluency. The media was then changed and supplemented with either 10 µM delta-9 tetrahydrocannabinol (THC) or 10µM cannabidiol (CBD), prepared in 100% methanol. Control culture dishes were mock treated with an equal volume of methanol. All treatments were performed in triplicate. Cell culture dishes were harvested after 24 h of drug exposure. Cell culture dishes were harvested after 24 h of drug exposure to capture protein changes and proteome remodeling [24]. The washed cell pellets were stored at −80 °C until use.

### 2.3. Sample Preparation for Mass Spectrometry Analysis

The sample preparation method was based on our previous work. The washed cell pellets were lysed in 8 M urea solution which also contained protease and phosphatase inhibitors, using a syringe lysis approach whereby samples were triturated ten times through a 1.5 cm long 21-gauge needle attached to a 1 mL syringe. Protein levels were estimated using a BCA protein assays. Reduction and alkylation of disulphide bonds was performed by incubation in 5 mM tris(2-carboxyethyl)phosphine for 30 min, followed by addition of 10 mM iodoacetamide for 30 min, and then addition of 10 mM DTT for 15 min to quench any remaining iodoacetamide. Chloroform-methanol precipitation was used to concentrate approximately 30 µg of protein which was then reconstituted in 100 µL of buffer consisting of 200 mM 3-[4-(2-Hydroxyethyl)piperazin-1-yl]propane-1-sulfonic acid pH 8.5. Protease digestion was performed at a 1:100 enzyme-to-protein ratio and consisted of two steps: overnight digestion with Lys-C at room temperature followed by 6 h digestion with trypsin at 37 °C. The resulting peptides were subsequently labeled using the TMTpro16 reagents for 60 min, with any remaining reagents quenched by the addition of 0.3% *v*/*v* hydroxylamine. To ensure near full, yet reagent-efficient, labeling with the TMTpro reagents, we labeled at a 2.2:1 TMTpro-to-peptide ratio. We ensured equal protein loading across all TMT channels at three stages. First, we performed a BCA protein assay at the lysate level, next, we adjusted for minor differences in precipitation efficiency by checking for an equal total TMT signal in all channels before sample pooling, and finally we normalized our data so as to equate total TMT signal across our final dataset.

The labeled peptides were combined and de-salted by purification with Sep-Pak C18 cartridges (Waters, Milford, MA, USA). The labeled peptide mixture was then fractionated using an Agilent 300 Extend C18 column (2.1 mm ID × 250 mm) and Agilent 1200 LC system. A gradient was developed over 15 min at a flow rate of 0.25 mL/min, from 5% acetonitrile in 10 mM ammonium bicarbonate (pH 8.0) to 35% acetonitrile in 10 mM ammonium bicarbonate (pH 8.0). A total of 24 fractions were collected for subsequent analysis by LC-MS3 [25,26].

### 2.4. Mass Spectrometry Data Acquisition and Analysis

The labeled peptide fractions were chromatographically separated on an Accucore C18 column (≈35 cm × 100 μm I.D.) (2.6 μm, 150 Å particle size; ThermoFisher Scientific, San Jose, CA, USA). A gradient was developed over 90 min from 94% buffer A (5% acetonitrile, 0.125% formic acid) and 6% buffer B (95% acetonitrile, 0.125% formic acid) to 60% buffer A, 30% B. Solvents were delivered using an Easy-nLC 1200 system, with the eluent directed into an Orbitrap Fusion Eclipse mass spectrometer (ThermoFisher Scientific, San Jose, CA, USA), and electro spray ionization conducted using 2.6 kV spray voltage.

Instrument parameters for spectral acquisition of MS1 scans in the Orbitrap included 350–1400 *m*/*z* mass range, automated gain control target 100%, resolution 60,000, and maximum injection time of 50 msec. Instrument parameters employed for spectral acquisition of MS2 scans in the ion trap included automated gain control target of 100%, rapid scan mode, isolation window of 0.7 Da, maximum injection time of 35 msec, and normalized CID collision energy of 35%. The final stage of the synchronous precursor selection (SPS) MS3 (DDA-SPS-MS3) method included online real-time database searching (RTS) enabled through the Thermo method editor. Instrument acquisition parameters for MS3 scans included an automated gain control target of 200%, isolation window of 1.2 Da, resolution 50,000, and maximum injection time of 200 msec. We used the DDA-SPS-MS3 method to help address the issue of interference in the MS2 spectra [27]. In this method, multiple MS2 fragment ions, which include the TMT label, are isolated and subjected to an additional fragmentation step, which removes any non-specific fragments (i.e., those belonging to another precursor) that were generated during the MS2 step. The SPS-MS3-based method provides higher accuracy compared to MS2 methods for TMT quantitation [28,29]. However, even with the added selectivity of SPS MS3, the accuracy can still suffer if non-specific fragments are selected for the MS3 step. To improve this, real-time search was implemented between the MS2 and MS3 scans. An MS3 scan is acquired only if the MS2 spectrum produces positive peptide identification [30]. We note that SPS-MS3 and real-time search enhances TMT quantification accuracy and proteome coverage, making it a powerful tool for quantitative proteomics.

All 24 fractions were analyzed twice, with a similar method that differed only in the compensation voltages (CVs) used for the FAIMS (high-field asymmetric ion mobility spectrometry) interface. The Thermo FAIMS Pro device was operated with default parameters (the inner and outer electrode were set at 100 °C, the dispersion voltage (DV) was set at −5000 V, and no additional gas was used). One CV set included −40 V, −60 V, and −80 V, while the second was −50 V and −65 V.

### 2.5. Bioinformatics Data Analysis

Peptide-to-spectrum matching was performed on mzXML files using the Sequest algorithm and a Uniprot human proteins sequence database (100,776 proteins, downloaded March 2022), supplemented with protein sequences of common laboratory contaminants. Searching was performed with a 50 ppm tolerance for precursor ions and a 0.9 Da tolerance for fragment ions. Oxidation of methionine residues (+15.9949 Da) was permitted as a variable modification, while static modifications were added to adjust for carbamidomethylation of cysteine residues (+57.0215 Da) and TMTpro modification of peptide N termini and lysine (+304.2071 Da). For initial testing of labeling efficiency, TMTpro modification was allowed as a variable modification.

The false discovery rate of PSMs (peptide-to-spectrum matches) was established using reversed database searching, and then adjusted to 1% [31]. Further filtering of PSMs was performed using linear discriminant analysis (LDA) [32], based on charge state, XCorr, ΔCn, peptide length, precursor mass accuracy, and number of missed cleavages. The final dataset produced a protein-level FDR of 1%.

A 0.003 Da window centered on the theoretical *m*/*z* of each reporter ion was scanned to enable reporter ion quantification of the most intense *m*/*z* detected. A summed signal-to-noise ratio of ≥100, across all labeled channels, was the minimum criteria for identified peptides to be retained for further analysis. Proteins quantitation values were produced by summing the previously filtered signal-to-noise values, and these were adjusted to be equal across experiments to compensate for any differences in protein loading. Finally, to allow quantitative comparisons across experiments, each protein identified was scaled so that the total within a TMTpro experiment summed to 100.

### 2.6. Further Analysis of Differentially Expressed Proteins

Analysis of variance (ANOVA) and fold change (FC) values between treated and control groups, calculated as the mean of the respective ratios, were used to identify differentially expressed proteins (DEPs). Statistically significant DEPs were those which past two criteria: *p* value less than 0.05 and FC greater than 1.20 for proteins increased in abundance, or less than 0.83 for proteins decreased in abundance [33]. Enrichr was used for gene ontology enrichment analysis at the biological process level and KEGG pathway enrichment analysis, and results were further evaluated using DAVID software (v2022q2) [34,35,36]. Cytoscape3.9 (https://cytoscape.org, accessed on 16 May 2022), with the STRING plug-in, was used to identify and visualize protein interaction networks based on both pathways and biological processes [37,38]. Proteins within a network that showed interactions with other proteins were used to identify gene hubs within networks based on a confidence cut-off of 0.5, as applied in the maximal clique centrality (MCC) algorithm in the CytoHubba plugin [39,40].

## 3. Results

### 3.1. Proteome Profiling of SH-SY5Y Cells and Significantly Differentially Regulated Proteins by CBD and THC Treatments

**Figure 1:**
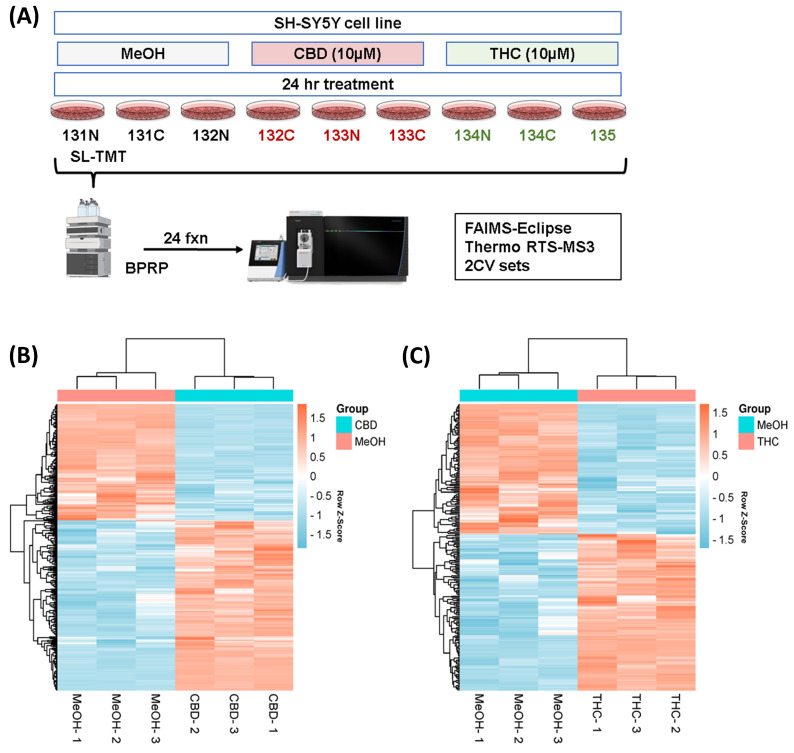
Hierarchical clustering for the two treatment groups. (**A**) Experimental arrangement and workflow overview. Heatmaps (with two-dimensional hierarchical clustering) showing the log-transformed ratios of differentially expressed proteins from (**B**) CBD vs. MeOH and (**C**) THC vs. MeOH treatment groups. BPRP—basic pH reversed phase; CBD—cannabidiol; MeOH—methanol; THC—delta-9 tetrahydrocannabinol.

We used sample multiplexing (TMTpro16) to profile the protein abundance alterations in the SH-SY5Y cell line following treatments with CBD and THC (as outlined in Figure 1A). A total of 9603 proteins were identified from the SH-SY5Y cell line in this study (Table S1). These proteins were quantified with multiple peptides at an initial protein FDR less than 1%. The number of differentially abundant proteins was higher in the CBD treatment group with 382 proteins, compared to THC treatment group with 238 differentially abundant proteins, according to ANOVA analysis (requiring both a *p*-value < 0.05 and the absolute value of fold change > 1.2). Hierarchical clustering analysis using Euclidean distance was applied to those differentially abundant proteins in both groups, and the results indicated similar expression patterns within the control and the CBD treatment groups (Figure 1C), as well as the control and the THC treatment groups (Figure 1C).

**Figure 2:**
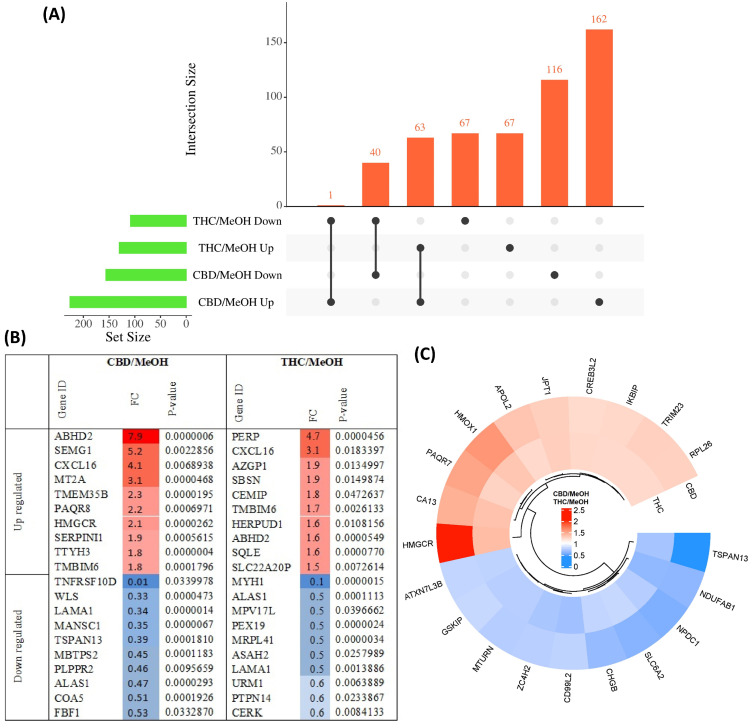
Overview of differentially abundant proteins. (**A**) UpSet plot showing the overlap of proteins with either an increased or decreased abundance in CBD and THC treatment groups. (**B**) Top 10 most significantly altered proteins increased and decreased in abundance in each group, with colour intensity corresponding to fold change, and (**C**) top 10 proteins with increased and decreased abundance shared between CBD and THC treatment groups.

The pairwise analysis between treatment groups and the control group yielded 156 proteins decreased in abundance and 226 proteins increased in abundance in response to CBD treatment, with respect to vehicle (MeOH) treatment. In addition, 108 proteins decreased in abundance and 130 proteins increased in response to THC treatment, also with respect to MeOH. Of these DEPs, 40 proteins decreased in abundance and 63 proteins increased in abundance were shared between CBD and THC groups, while 134 proteins were altered in abundance only by THC treatment and 278 proteins were altered in abundance only by CBD treatment (Figure 2A). Only one protein increased in abundance in CBD treatment and decreased in THC treatment; fatty acyl-CoA reductase 1 (FAR1), which plays a role in providing fatty alcohols needed to create ether bonds in the synthesis of ether glycerophospholipids. DEPs are listed in Table S2 for CBD and Table S3 for THC. The top 10 proteins with respect to a decrease and increase in abundance in each group, sorted by *p*-value, are shown in Figure 2B, while the top 10 proteins with respect to decrease or increase in abundance and that were also shared between both groups, are shown in Figure 2C.

The protein interactions between these DEPs and the top identified related KEGG pathways of both groups are illustrated in Figure S1, with the data used to produce this figure presented in Supplementary Table S4. This analysis showed several proteins that increased in abundance in response to the CBD treatment were involved in ECM–receptor interactions, whereas a number of proteins involved in steroid biosynthesis increased in abundance when cells were treated with THC. Proteins involved in oxidative phosphorylation and protein processing in the ER decreased in abundance when cells were treated with CBD, but increased in abundance following treatment with THC.

### 3.2. Pathway Classification of the Differentially Expressed Proteins

**Figure 3:**
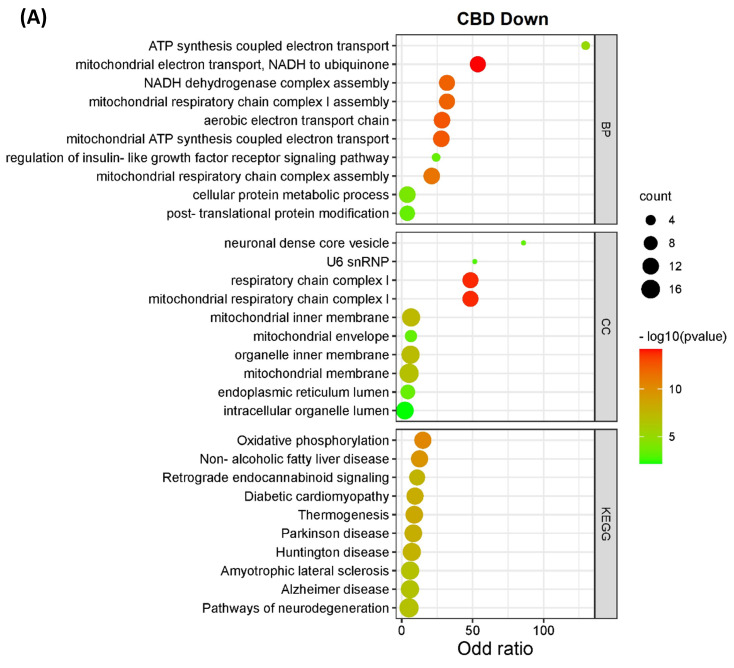

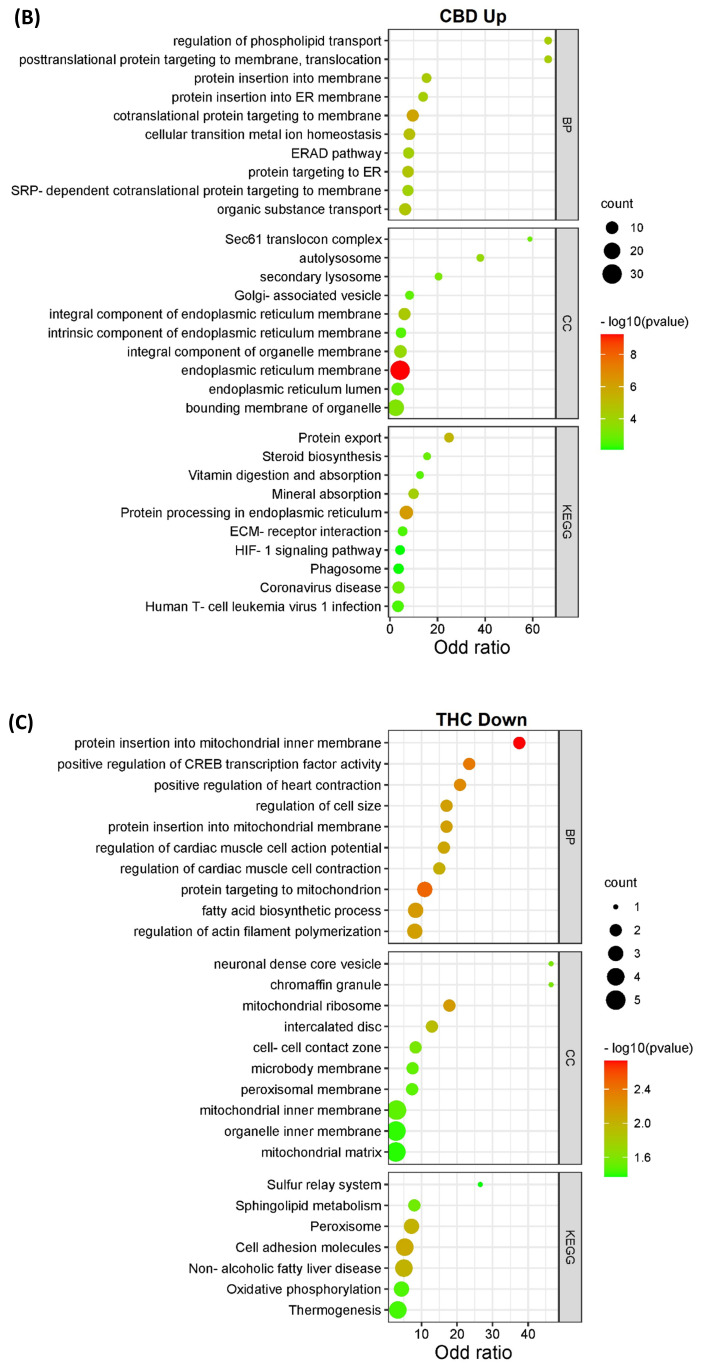

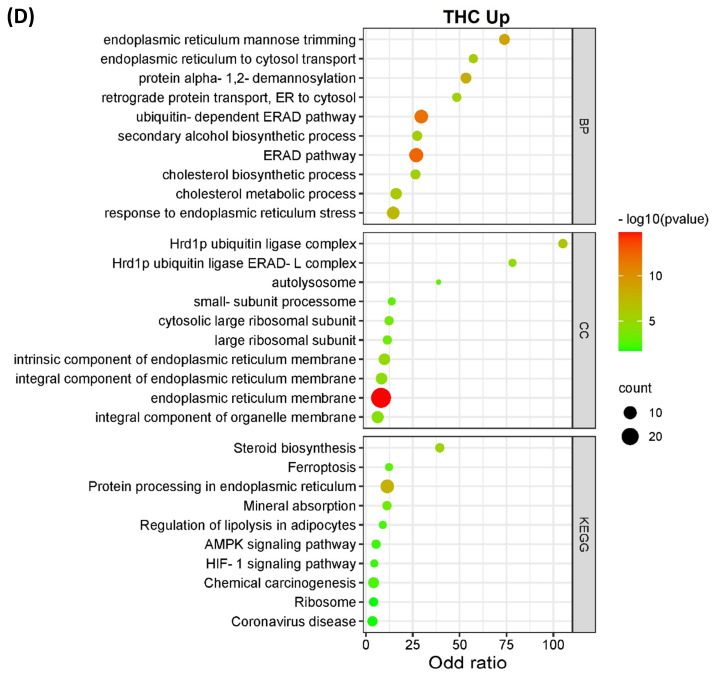
KEGG pathways, biological processes, and cellular components of differentially expressed proteins. The bar charts illustrate the top 10 most significantly regulated categories for KEGG pathway, biological process, and cellular components separated by treatment and direction. The categories are listed for (**A**) decreased and (**B**) increased proteins in the CBD treatment group, as well as (**C**) decreased and (**D**) increased proteins in the THC treatment group.

The DEPs from both CBD versus MeOH and THC versus MeOH groups were subjected to KEGG pathway and GO enrichment analysis to identify molecular mechanisms corresponding to beneficial or toxic effects of these cannabinoids. Taken together, the analyses yielded several pathways, biological processes, and cellular components affected by CBD and THC. The top 10 most significant KEGG pathways, biological processes, and cellular components enriched by DEPs are shown in Figure 3. Specifically, the categories listed include proteins decreased (Figure 3A) and increased (Figure 3B) in abundance in the CBD treatment group, as well as those decreased (Figure 3C) and increased (Figure 3D) in abundance in the THC treatment group.

**Figure 4:**
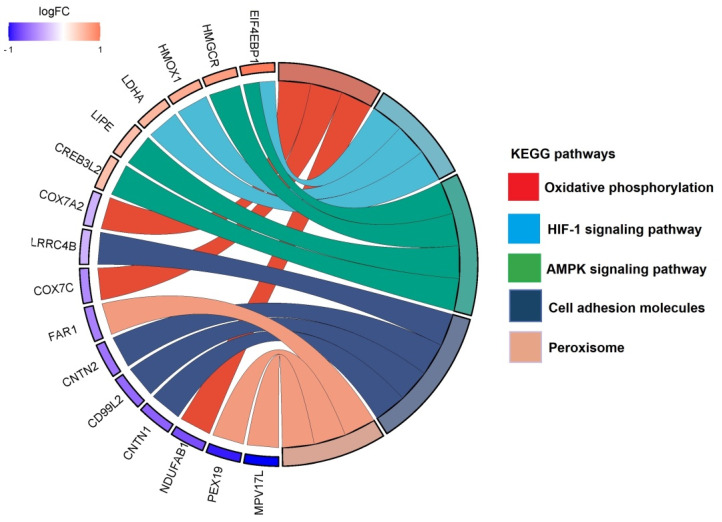
Top identified KEGG pathways with associated proteins in the THC treatment group compared to control. Chord diagram illustrating the top KEGG pathways altered in our dataset and the associated proteins.

Interestingly, the data from all enriched KEGG pathways, biological process, and cellular components analyses revealed that the mitochondria and endoplasmic reticulum may be among the main targets of both CBD and THC. Oxidative phosphorylation, which is a major function of mitochondria, was significantly influenced by CBD, but less so by THC with less DEPs identified (Figure 4), while other enriched pathways, biological processes, and cellular components showed that mitochondria may have been affected by treatment with both cannabinoids. In addition to the pathways shown in Figure 3, other significant results from this functional analysis included HIF-1 signaling pathway, ERAD pathway, steroid biosynthesis, and ferroptosis markers were shown to be altered in abundance in both CBD- and THC-treated groups (Table S4 and Figure S1). Regarding the differences between CBD and THC treatment groups, CBD showed a decreased abundance of proteins involved in pathways related to neurodegenerative diseases, and an increased abundance in the complement and coagulation cascades, p53 signaling pathway, necroptosis, and ECM–receptor interactions.

Conversely, THC showed down-regulation of different pathways such as cell adhesion molecules and peroxisome, and up-regulation of pathways involved in AMPK signaling and lipid metabolism.

### 3.3. Protein-Protein Interaction (PPI) Analysis

**Figure 5:**
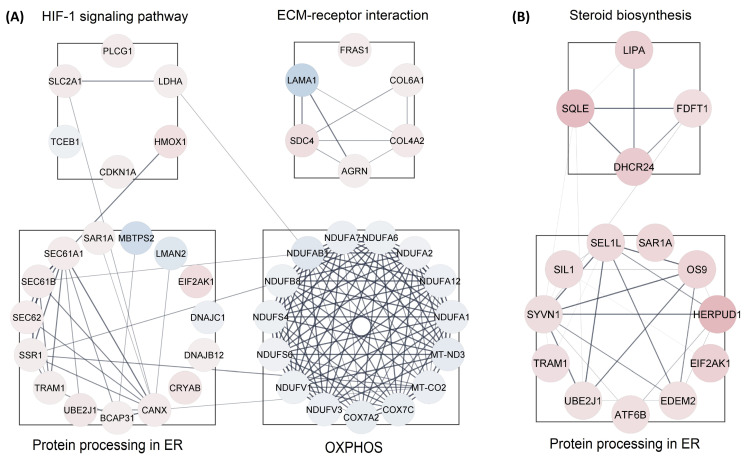
Protein-protein interactions for enriched pathways. Interactions maps of up-(red) and down-(blue) regulated proteins for the (**A**) CBD and (**B**) THC treatment groups. ER—endoplasmic reticulum; OXPHOS—oxidative phosphorylation.

**Figure 6:**
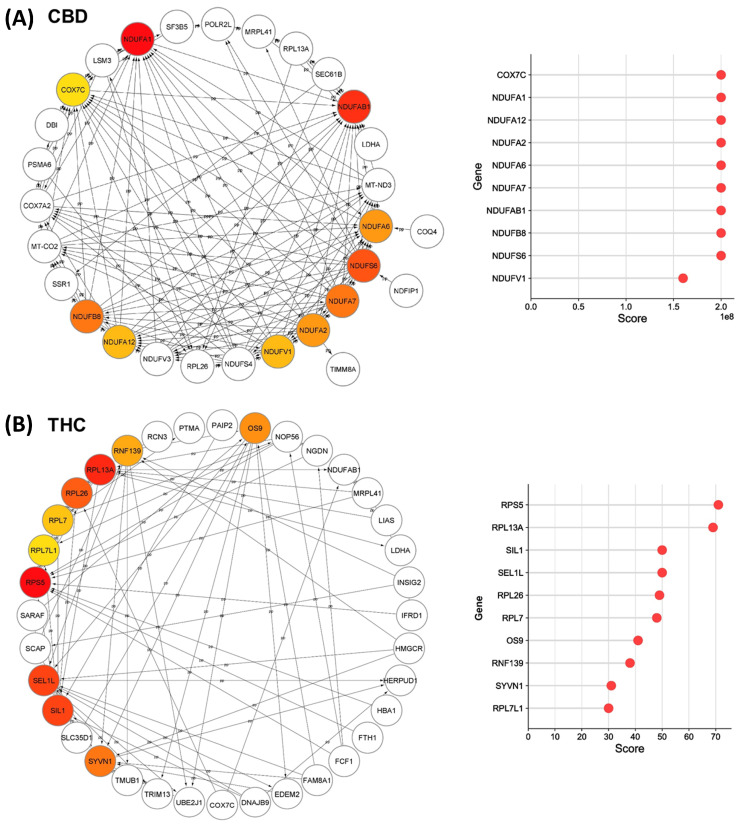
Top-ranked hub genes within the identified network in Cytoscape. Network diagrams illustrate the top 10 hub genes in the identified network for (**A**) CBD and (**B**) THC treatment groups, highlighted with red to yellow based on their yielded maximal clique centrality (MCC) score that are shown in the right plots.

We analyzed all DEPs from each treatment group to interrogate protein networks using KEGG pathways. To summarize these results, we depicted PPIs of the DEPs from both treatment groups within the identified network using circular layouts in Figure 5. Our analysis revealed protein networks that impact four pathways in the CBD treatment group, specifically, oxidative phosphorylation, HIF-1 signaling pathway, ECM–receptor interaction, and protein processing in the endoplasmic reticulum (Figure 5A). Meanwhile, two pathways were impacted in the THC treatment group: protein processing in the endoplasmic reticulum and steroid biosynthesis (Figure 5B). In addition, hub gene analysis resulted in identification of the top 10 hub genes among all DEPs in each treatment group [41]. Interestingly, these genes were related to the oxidative phosphorylation chain in CBD treatment group, while hub genes in THC group were mostly ribosomal proteins (Figure 6).

Our data also showed down-regulation of the oxidative phosphorylation pathway and up-regulation of protein processing in the endoplasmic reticulum. Oxidative phosphorylation is the main source of energy production within the cell. Dysregulated oxidative phosphorylation pathways are considered key drivers of pathological events in various diseases. This pathway was significantly enriched by 13 proteins decreased in abundance in CBD treatment. However, only three proteins in this category were decreased in abundance in the THC treatment group, and as such, protein-protein interaction analysis was not possible for the THC treatment group. Protein processing in endoplasmic reticulum was another commonly impaired pathway between both treatment groups, enriched by 12 and 11 proteins increased in abundance in CBD and THC treatment, respectively. Four of these proteins were shared between the two treatment groups: SAR1A, TRAM1, UBE2J1, and EIF2AK1 (Figure 5). The up-regulation of HIF-1 signaling pathway, steroid biosynthesis, and ECM–receptor interaction were among the most significantly dysregulated pathways observed. The HIF-1 signaling pathway was enriched by 5 and 3 proteins, while steroid biosynthesis was enriched by 3 and 4 proteins in CBD and THC groups, respectively. In addition, the ECM–receptor interaction was significantly enriched in the CBD group by 5 proteins increased in abundance (Figure 5).

## 4. Discussion

CBD and THC, the most abundant cannabinoids in the cannabis plant, have garnered increasing interest as therapeutic agents for use against several diseases including neurodegenerative disorders, diabetes, and cancer [2]. Their beneficial effects have been reported to be through several mechanisms suggesting anti-inflammatory, immunosuppressive, anti-proliferative, and neuroprotective effects [8]. The urgent need for developing new and more-effective drugs, especially for such devastating diseases as listed above, has led to an ever-increasing body of research on exploring their therapeutic effects. Not surprisingly, more studies have been presented on the beneficial effects of new cannabinoid-derived agents than their adverse effects. However, detrimental effects are an inseparable part of drug treatments and should be thoroughly investigated. These side effects must be weighed against their benefits for a given pathological condition. In this regard, CBD has shown a wider margin of safety than THC and thus has received greater attention for drug development [2]. However, several complications have been associated with CBD use, including neurodevelopmental toxicity, embryo-fetal mortality, hepatotoxicity, reproductive system toxicity, and hypotension in animals studies, as well as CBD-induced drug–drug interactions, hepatic abnormalities, diarrhea, fatigue, vomiting, and somnolence in human studies [42]. Besides expanding its therapeutic use, the consumption of cannabis as a recreational product due to its psychotropic effect has been increasing, especially among younger age groups [43]. Therefore, comprehensive studies are needed to evaluate toxicity of cannabis-derived compounds.

In the present study, the effect of both THC and CBD on the proteome of the SH-SY5Y cell line has been investigated to explore potential mechanisms underlying their toxicity and therapeutic properties. The SH-SY5Y cell line is a human-derived cell line that is widely used in scientific research and is used as an in vitro model for neuronal function and differentiation [44]. Although we used undifferentiated cells, SH-SY5Y cells can be differentiated into several different phenotypes depending on the treatment. Commonly used differentiation agents include phorbol esters and retinoic acid, growth factors such as brain-derived neurotrophic factor, nerve growth factor, and neuregulins [45]. Future experiments could potentially incorporate differentiation with various strategies, and investigation of the proteome remodeling due to THC and CBD treatment using the workflows discussed herein.

In vitro studies have suggested that CBD can have neuroprotective effects on SH-SY5Y cells at concentrations ranging from 0.1 to 10 μM [46,47,48,49]. We used a concentration of CBD (10 µM) that was observed to not reduce cell viability while still promoting autophagy in SH-SY5Y cells [49]. The same concentration was used in a study investigating miRNA in the context of CBD-treated cells [50]. Likewise, THC has been used at various concentrations in in vitro studies [51,52,53,54]. We selected 10 µM as the concentration of THC as it has been found previously to be neuroprotective in cell culture models [52] and well-tolerated in SH-SY5Y cells [54].

The comparison between cannabinoid treated and control groups showed a larger proportion of DEPs in response to CBD treatment, with 382 DEPs, compared to the THC treatment with 238 DEPs. The criteria used in this study to define a DEP involved both a *p*-value cut-off and a fold change cut-off [55,56], which is sufficiently strict to ensure false discovery rates are appropriately controlled [33,57], while also not increasing the false negative rate, which is an issue with multiple testing corrections in experiments of this scale [58]. This also minimizes the need for confirmatory experiments such as Western blotting of selected proteins, which are generally not statistically significant. Experimental confirmation of DEPs of interest for further study could be provided by the use of multiplexed reaction monitoring assays [59].

Further analyses of DEPs revealed common and distinctly altered KEGG pathways, biological processes, and cellular components in SH-SY5Y cells following treatment with CBD and THC. Our results showed down-regulation of mitochondrial and endoplasmic reticulum genes in both the treatment groups, and these effects were more pronounced in CBD treatment group compared to the THC group. Previous evidences on the effects of CBD on the electron transport chain (ETC) complexes have, to date, been controversial [60]. While evidence indicated the protective effects of CBD on mitochondria and subsequently ETC in animals and primary cell lines [61,62], inhibitory effects of CBD on mitochondrial function has also been reported in different cell lines, especially cancer cells. For instance, both acute and chronic intraperitoneal administration of CBD showed increased activity of ETC complex I, II, III, and IV in rat brain tissues [62].

In addition, CBD showed neuroprotective features in oxygen-glucose-deprived hippocampal neurons through restoring mitochondrial respiration [61]. Conversely, in vitro treatment of isolated pig brain mitochondria with THC and CBD showed decreased ETC complex I, II/III, and IV activity [63,64]. In addition, the inhibitory effects of CBD on mitochondrial function were suggested to be through inhibition of voltage-dependent anion channel 1 (VDAC1) conductance, which then limits the amount of substrate available for cellular respiration and leads to cell death in microglia cells [65]. Other studies on different cancer cells also suggest inhibitory effects of CBD on mitochondria. Such effects are manifested as significantly decreased basal respiration rate and overall ATP production, which is associated with decreased cell proliferation and tumor growth [66,67]. Different mechanisms have been suggested to prevent the toxic effects of CBD on mitochondria, such as altering mitochondrial ability for Ca^2+^ homeostasis. One such possibility is through inhibiting VDAC channel conductance and stimulating these channels to become highly permeable to Ca^2+^, which would lead to mitochondrial Ca^2+^ overload, and in turn result in stable mitochondrial transition pore formation and cell death [68]. The inconsistencies among published results can be due, in part, to the wide range of treatment dosages, the cell and tissue types used, and administration modalities employed. Herein, our results indicated that CBD induced a significant down-regulation of mitochondrial ETC complexes, especially complex I, in SH-SY5Y cells. Regarding the effects of THC on mitochondria, the lower number of DEPs in the THC group in our study did not indicate a significant difference in mitochondrial oxidative phosphorylation. However, the down-regulation of mitochondrial genes and proteins associated with oxidative phosphorylation is common in proteomic and genomic studies on animal brain samples following exposure to THC [15,69,70,71]. Moreover, down-regulation of ETC-associated proteins and increased oxidative stress is present in almost all studies [72,73,74].

Another significant finding of this study was increased abundance of proteins associated with the ER in both the CBD and THC treatment groups. The ER plays an essential role in lipid and protein synthesis and maturation, calcium homeostasis, and protein folding. Intermediate ER stress acts as a defense mechanism to increase protein folding in an attempt to restore homeostasis, but severe or prolonged ER stress may result in initiation of apoptosis, which has been shown to be a mechanism underpinning the toxic effect of CBD against cancer cells [75]. Our results showed up-regulation of an IRE1-mediated unfolded protein response (UPR) biological process, and the increased abundance of proteins involved in UPR, such as ATF6B, especially in the CBD-treated group. In addition, the data showed that proteins with roles in the ER-associated degradation (ERAD) pathway were increased in abundance. ERAD eliminates misfolded, unassembled, or metabolically regulated proteins by the cytosolic ubiquitin proteasome system (UPS) to alleviate unfolded protein accumulation and enhance protein folding in the ER [75]. Collectively, activation of these processes indicated the activation of ER-UPR. In line with our results, up-regulation of ER-UPR has been reported in lung epithelial cells treated with CBD, and it has also been suggested that ER-UPR and ERAD are potential defense mechanisms against COVID-19 replication [76].

Results of this study also showed up-regulation of the HIF-1 signaling pathway, which is a transcription factor and a key regulator of oxygen homeostasis. Activated HIF-1 plays a crucial role in response to pathological conditions through transcriptional activation of over 100 downstream genes involved in different biological processes required for cell survival such as glucose metabolism and angiogenesis [77,78]. Although HIF-1 was not identified in our results, its downstream target genes were found to be up-regulated, including LDHA, CDKN1A, SLC2A1, HMOX1, and PLCG1. CBD has been shown previously to decrease angiogenesis and stemness of breast cancer cells through down-regulation of HIF-1 [79]. HIF-1 is closely associated with mitochondrial dysfunction. As such, mitochondrial dysfunction may lead to HIF-1 signaling pathway up-regulation which, in turn, shifts glucose metabolism from oxidative phosphorylation to the glycolytic pathway, to maintain metabolic homeostasis. Conversely, HIF-1 signaling pathway up-regulation may also explain mitochondrial dysfunction [77].

Steroid biosynthesis is another pathway that was enriched in both the CBD and THC treatment groups. Recent studies have shown that steroid hormones can activate UPR in normal and cancer cells and promote cancer cell death [80,81]. In addition, CBD has been shown to increase the expression of estrogen receptor beta (Erβ), which is a key target for estrogen receptor-positive (ER+) breast cancer treatment [82]. Another significantly altered pathway was the ECM–receptor interaction pathway that was shown to be up-regulated only by CBD treatment, but not by THC treatment. Interactions between ECM and cells are crucial for cell adhesion, migration, differentiation, proliferation, and apoptosis [83,84]. Up-regulation of proteins was also observed in pathways reported to be associated with tumor shedding, adhesion, degradation, movement, hyperplasia, and poor overall survival [85,86]. For instance, increased expression of COL6A1, SDC4, FRAS1, AGRN, and COL4A2 has been observed in different cancer types and reported to be associated with metastasis and poor outcomes in patients [87,88,89,90,91]. Higher expression of COL6A1 has also been observed in glioblastoma multiforme compared to normal brain tissues and was associated with a poor prognosis [92]. Interestingly, SDC1 also plays a key role in preventing cellular senescence via modulating FGFR1 endocytosis, which is critical for various cellular processes such as differentiation, growth, proliferation, and survival [93,94,95].

## 5. Conclusions

The results of the present study reveal the biological pathways affected by CBD and THC treatment in the SH-SY5Y cell line and represent a valuable resource of protein abundance profiles for future studies. In addition, our findings suggest an impairment of mitochondria and the endoplasmic reticulum as the major targets affected by CBD and THC treatments. These treatments may result in mitochondrial dysfunction and endoplasmic reticulum stress, which drive downstream effectors. This information will provide a foundation for future animal and clinical studies, and this dataset can be mined further to investigate the potential role of CBD and THC in different biological contexts.

## 6. Limitations

This study contains some inherent limitations because it involves bottom-up proteomics analysis in which proteins are first enzymatically digested into peptides. The authors acknowledge that this approach dictates that the manuscript contains little information on the state of intact proteoforms because that is lost during the digestion stages. Similarly, this manuscript and these experiments are not focused on proteins species, with no attempt at enrichment of particular post-translational modifications. The focus of the study was individual gene products and how these were altered in abundance in cells in response to treatment with cannabinoids.

The identification and quantitation of proteins changed in abundance in cell lines in response to treatment with cannabinoids demonstrates once again the power of procurement analysis in unbiased discovery, and provides an excellent foundation for further interrogation and investigation of the large-scale dataset produced. Qualitative comparative proteolytic analysis studies such as this are foundational building blocks in our understanding of how the overall protein and complexity in different cells respond differently to external treatments. Cellular proteomes are complex systems, and describing the components of a complex system is an essential first step in understanding how that system functions.

## Data Availability

Mass spectrometric data are available via ProteomeXchange with identifier PXD038121.

## Supplementary Materials

The following supporting information can be downloaded at: https://www.mdpi.com/article/10.3390/proteomes11040036/s1: Table S1: Proteins identified in the dataset. Columns include: Uniprot protein ID, gene name, protein description, number of peptides matched to these proteins and the TMT signal-to-noise values for all 9 samples. Table S2: Differentially expressed proteins for CBD treatment. Columns include: Uniprot protein ID, gene name, protein description, the CBD/MeOH ratio, and the associated *p*-value. Table S3: Differentially expressed proteins for THC treatment. Columns include: Uniprot protein ID, gene name, protein description, the THC/MeOH ratio, and the associated *p*-value. Table S4: enriched KEGG pathways, biological processes and cellular components in the CBD and THC treated cells. Figure S1: summary of the comparison between CBD and THC treatment groups, including protein—protein interactions.
