# Supplementary material for: Proteome-Wide Profiling Using Sample Multiplexing of a Human Cell Line Treated with Cannabidiol (CBD) and Tetrahydrocannabinol (THC)
Source: Proteomes. 2023 Nov 2;11(4):36. doi: 10.3390/proteomes11040036 (PMC10661330; doi:10.3390/proteomes11040036)
Supplement: Supplementary file 1 [file Abyadeh at al Figure S1.pdf › Abyadeh at al Figure S1.pdf]

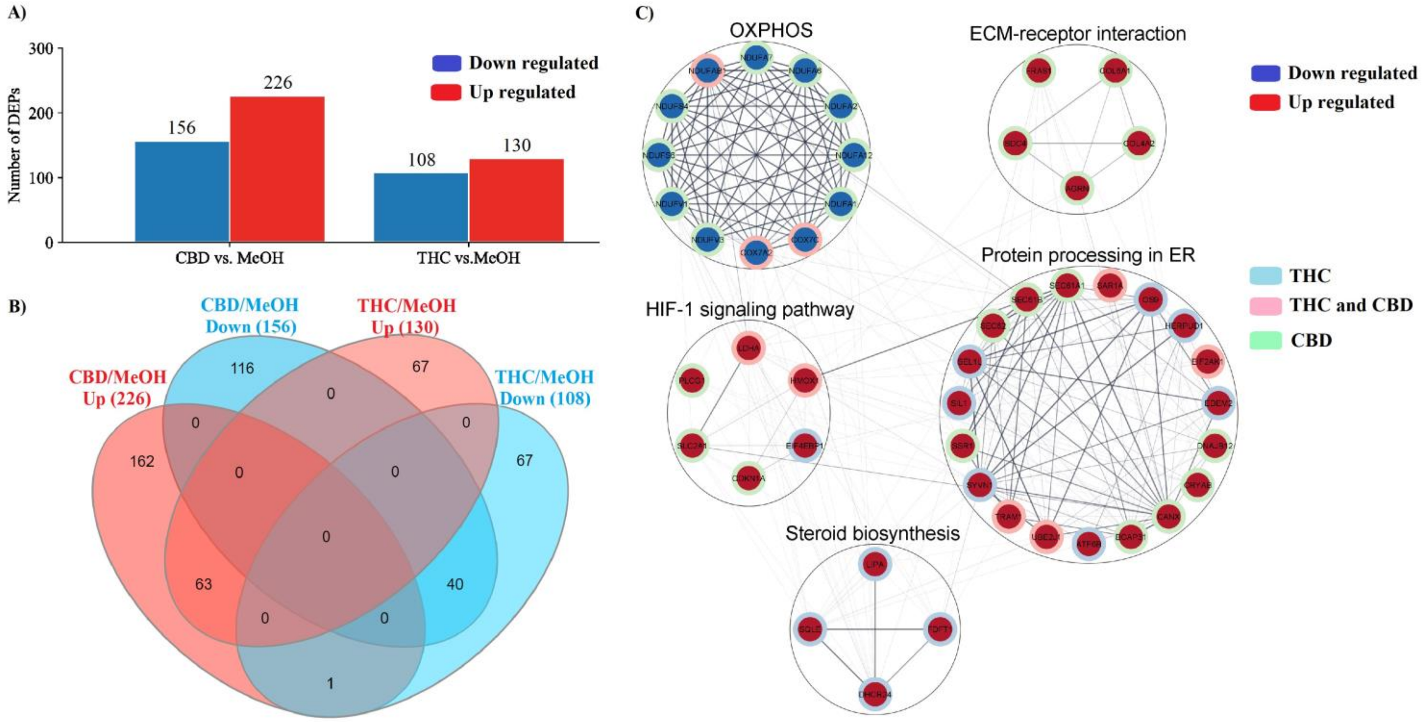

Supplementary figure: A summary of the comparison between the groups treated with CBD and THC. A) The number of proteins that were down-regulated (in blue) and up-regulated (in red) in each treatment group compared to the control group treated with MeOH. B) The number of proteins that were shared or distinct between the different treatment groups. C) A visualized PPI network of the down-regulated (in blue) and up-regulated (in red) proteins from the CBD (in green), THC (in blue), and shared DEPs (in pink) between both groups using Cytoscape.
